# Factors associated with stunting and underweight indices among children 0–3 years in Nairobi, Kenya: a cross-sectional study

**DOI:** 10.3389/fnut.2026.1793821

**Published:** 2026-05-11

**Authors:** Eunice Njoroge, Kevinson Mwangi, Esther Jebor Chongwo, Martha Kaniala, Kevin Wekesa, Isaack Lihanda, Vibian Angwenyi, Caroline Ngunu, Judy Macharia, Naomi Kigani, Rachel Odhiambo, Margaret Kabue, Japheth Adina, Amina Abubakar

**Affiliations:** 1 Institute for Human Development, The Aga Khan University, Nairobi, Kenya; 2 Department of Health, Nairobi City County Government, Nairobi, Kenya; 3 Wellcome Trust Research Programme, Centre for Geographic Medicine Research (Coast), Kenya Medical Research Institute, Kilifi, Kenya

**Keywords:** child, Kenya, Nairobi, stunting, undernutrition, underweight, urban-setting

## Abstract

**Background:**

Child undernutrition remains one of the most pressing global public health challenges, particularly in low and middle-income countries (LMICs). Most studies focus broadly on children under five, potentially overlooking the unique vulnerabilities affecting children in the first 1,000 days of life. This study investigated potential factors associated with stunting and underweight malnutritional presentation among children aged 0–3 years in Nairobi City County, Kenya.

**Methods:**

We conducted a cross-sectional study of 2903 caregiver-child dyads living in Nairobi City County to assess stunting [Height-for-Age *Z*-scores (HAZ)] and underweight [Weight-for-Age *Z*-scores (WAZ)] using the World Health Organisation (WHO) growth standards among 0–3-year-olds. Child, caregiver, and household characteristics were included in the linear mixed effects regression models of factors associated with the study outcomes.

**Results:**

The mean (standard deviation) HAZ and WAZ were −0.90 (1.5) and −0.38 (1.3), respectively. Approximately 22.0% of children in the study were stunted and 10.0% underweight (< −2 *Z*-score). Common factors associated with stunting and underweight in multivariable models were older age (β = −0.04 and β = −0.01, respectively, both *p* < 0.001), male gender (β = −0.30 and β = −0.22; Reference = Female, respectively, both *p* < 0.001), low birth weight (β = −1.19 and β = −0.94), respectively, both *p* < 0.001, and living in informal settlements (β = −0.15, *p* = 0.010 and β = −0.14, *p* = 0.008). Additionally, severe household food insecurity (β = −0.47, *p* = 0.004; Reference = Secure) was associated with stunting, while high wealth index (β = 0.16, *p* = 0.018; Ref = Low wealth index) and history of hospitalization (β = −0.17, *p* = 0.032) were associated with underweight.

**Conclusion:**

The high burden of stunting and underweight in this setting signals a serious public health risk and necessitates tailored interventions targeting individual and household-level vulnerabilities.

## Background

Child undernutrition remains one of the most pressing global public health challenges, particularly in low- and middle-income countries (LMICs). Stunting (low height-for-age) and underweight (low weight-for-age) are critical indicators of chronic and acute malnutrition, respectively (1). In 2022, an estimated 149 million children under five were stunted, 37 million were underweight, and 45 million were wasted worldwide (2). Nearly half of all deaths among children under five are linked to undernutrition, with the majority occurring in LMICs (3). The consequences of undernutrition are both immediate and long-term, including increased vulnerability to infections, impaired cognitive development, poor academic performance, reduced economic productivity in adulthood, and increased risk of non-communicable diseases later in life (3).

In Sub-Saharan Africa (SSA), the burden of undernutrition remains alarmingly high despite sustained global and regional efforts. The region accounts for nearly one-third of the world's stunting burden in children, with a stunting prevalence of about 30%, and underweight rates reaching up to 20% in certain countries (4). The high burden of undernutrition is driven by a complex interplay of factors such as poverty, food insecurity, inadequate access to healthcare, poor sanitation, and suboptimal infant and young child feeding (IYCF) practices (5, 6). Urban areas in SSA present a nutritional paradox: while urbanization is associated with better access to services and health outcomes, children living in informal settlements within these urban centers are disproportionately affected by both undernutrition and overnutrition (7). Progress toward Sustainable Development Goal (SDG) 2, which seeks to end hunger and all forms of malnutrition by 2030, has been slow across SSA, with many countries experiencing stagnating or a reversal of gains (8). Recent reports indicate that no country in SSA is currently on track to meet the SDG nutrition targets (8). Although there has been a gradual reduction in the proportion of stunted children under five in Africa from 38% in 2000 to 29% in 2020, the current levels are still far from the 2025 global target of 16%, and the 2030 target of 11% (8, 9).

Existing literature across SSA and including a study in Kenya has identified multiple determinants of childhood undernutrition, including maternal education, household income, food availability, suboptimal breastfeeding and complementary feeding practices, and access to health services (10). However, notable knowledge gaps persist because most studies examine children under five as a single group, overlooking the unique vulnerabilities within the first 3 years of life, a critical development window (11). Additionally, although stunting and underweight share overlapping risk factors, they arise form distinct biological and environmental pathways, yet studies in Kenya have examined undernutrition outcomes collectively (5). Stunting reflects long-term, cumulative deprivation, whereas underweight captures both chronic and acute influences, potentially responding differently to socioeconomic, caregiving, and health-related factors (12). There is limited evidence on determinants of stunting and underweight from urban Kenyan settings, where environmental and socioeconomic exposures differ from rural populations. Furthermore, existing studies in Nairobi focus on sub-populations, constraining the representativeness of findings to the wider population. For instance, research conducted among under-five children in informal urban settlements in Nairobi has documented a high burden of malnutrition, but its settlement specific focus limits broader urban generalizability (13).

Anchoring this study within the United Nation Children's Fund (UNICEF) conceptual framework for the determinants of child undernutrition (14), the study examined how immediate determinants (e.g., child age, history of illness, and birthweight), underlying determinants (e.g., caregiver-related care practices like antenatal care (ANC) attendance and household food insecurity), and basic structural determinants (e.g., socioeconomic status and settlement type) interact to shape early childhood growth outcomes. This study addresses these gaps by investigating multilevel factors associated with stunting and underweight among children aged 0–3 years in Nairobi City County, Kenya. By focusing on this critical developmental window and examining an urban population, the study aims to generate evidence that can inform targeted, context-specific interventions and policy frameworks to combat child malnutrition in rapidly urbanizing environments.

## Methods

### Study design and setting

This was a community-based cross-sectional study, nested in a broader project aimed at evaluating the status of early childhood development in Nairobi City County, Kenya. Nairobi City County is one of Kenya's 47 counties (subnational governments) and hosts the country's capital. Geographically located on latitude 1.29° S and longitude 36.82° E, the county had a population of approximately 4.4 million people in 2019, with an estimated annual intercensal population growth rate of 3.4% between 2009 and 2019 (15). Nairobi County is highly heterogeneous, with a population characterized by significant disparities in wealth and access to health services (16).

### Participants, sampling, and data collection procedures

The study targeted primary caregivers of children aged 0–3 years residing in Nairobi City County. The respondents were either the child's parent or an adult caregiver living with the child full-time in cases where the parents were absent or deceased; English/Swahili language speakers; had capacity to provide written informed consent, and were available for a face-to-face interview.

Sampling was done across all 17 sub-counties in Nairobi City County to enhance the representativeness of findings. The overall sample size (*n* = 2,903) was distributed across the 17 sub-counties in proportion to their population sizes, based on estimates from the Kenya National Bureau of Statistics (KNBS) (17). Within each sub-county, sample size was further stratified by wealth categories (high, middle, low), proxied by neighborhood, to reflect the county's socioeconomic diversity.

Households were identified with the assistance of Community Health Promoters (CHPs), who maintain routine household registers within their catchment areas. In each selected sub-county and settlement type, CHPs used these registers to identify households with children aged 0–3 years. Eligible households were randomly sampled and approached until the target sample size for each stratum was attained. Proportional allocation across sub-counties and stratification by settlement type and wealth categories were employed.

The study was conducted in collaboration with the Nairobi City County personnel. As such, the research team leveraged the existing county and subcounty health administrative structure to facilitate community entry and participants identification. These structures consisted of community health assistants (CHAs), community health promoters (CHPs), and their supervisors at sub-county and county levels.

Data collection was done between September and October 2024 by a team of 42 trained field enumerators and six field supervisors. Prior to data collection, the team participated in a 2-week training, which covered research ethics, survey administration, bias mitigation, and field testing of the survey instrument.

Eligible respondents were approached in their households where detailed study information giving and written informed consent was obtained. The survey interviews were conducted in quiet and private location to maintain confidentiality, while the child's anthropometric assessments were performed in a central location after the interviews. Data were collected using encrypted mobile tablets programmed with Open Data Kit (ODK) forms and were securely uploaded to a server with restricted access to authorized research personnel.

### Measures and definitions

#### Outcomes

The study had two outcome variables: stunting, as defined by height-for-age (HAZ) *z*-scores, and underweight, as defined by weight-for-age (WAZ) *z*-scores. HAZ and WAZ scores were generated based on the WHO Growth Reference Standards (2006) (18), using the “zscorer” package in R statistical software (19). We used the cutoff *z*-scores < 2 SD below the reference population median to describe the proportion of children in our sample who were stunted or underweight.

Anthropometric measurements taken from children were weight, length/height, and Middle Upper Arm Circumference (MUAC), following WHO-recommended procedures (20). Weight was measured using mobile-calibrated SECA digital scales to the nearest 0.1 kg. Length/height was measured using a SECA length board to the nearest 0.1 cm, with recumbent length taken for children below 2 years and standing height for those above 2 years. MUAC was measured on the left arm using a color-coded MUAC tape to the nearest 0.1 cm.

#### Explanatory variables

Explanatory variables included in this study were child, primary caregiver, and household-level characteristics informed by existing literature (21). Child-level variables included age, gender, birth weight and history of hospital admission. Caregiver-level variables consisted of age, marital status, education level, antenatal care (ANC) attendance, place of delivery, self-reported pregnancy history and related complications, mental health status including depression and anxiety. The Patient Health Questionnaire-9 (PHQ-9) (22), and the Generalized Anxiety Disorder-7 (GAD-7) (23), were tools used to assess caregiver depressive and anxiety symptoms, respectively. Both tools have been validated for use in Kenya (24), and demonstrated good internal consistency for our sample (Cronbach's alpha = 0.81 and 0.85, respectively).

Household-level variables included wealth index, settlement type (formal vs. informal), number of children ≤ 3 years, food insecurity status, presence of a child with disability in households surveyed, and the quality of home environment. The wealth index was assessed using variables from the Demographic Health Survey (DHS) programme and analyzed through a principal component analysis (25), categorizing households into low, middle, or high wealth index status. The Household Food Insecurity Access Scale (HFIAS) was used to evaluate a household's food security status (26). The nine-item tool scores range from zero to 27, with higher scores indicate greater food insecurity status, and households classified as either food secure, mildly insecure, moderately insecure, or severely insecure. The HFIAS demonstrated acceptable internal consistency (Cronbach's alpha = 0.91). The quality of home and family environment in promoting child development were assessed using an adapted version of Home Screening Questionnaire (HSQ), our version has 22 items scored as binary or multiple choice (27). Responses were summed up to generate a total score (range: 0–22) with higher scores indicating more stimulating environments. HSQ showed an acceptable internal consistency in our sample (Cronbach's alpha = 0.70).

#### Statistical power calculations

The present study was a part of a larger study originally designed to assess the state of early child development in Nairobi City County. We therefore conducted a *post-hoc* power analysis for the present analysis. The intraclass correlation from our unconditional multilevel model was 0.02, suggesting minimal clustering of stunting and underweight outcomes within sub-counties. After accounting for the design effect, the study had 85.6% power to detect small effects (Cohen's *f*^2^ = 0.035) for up to 18 predictors, at the individual-predictor level, at the 5% significance level.

#### Statistical analyses

All statistical analyses were conducted in R statistical software version 4.4.1, “Race for Your Life” (28). Continuous and categorical variables were summarized using means (SD) and frequency (%), respectively. Univariable and multivariable linear mixed effects regression models were used to assess factors associated with HAZ and WAZ scores. The models included a random intercept for subcounty to account for the potential clustering of stunting and underweight outcomes of children within subcounties in Nairobi City County. The continuous outcome variables were preferred over a binary categorization, as it would lead to information loss. HAZ and WAZ scores followed a normal distribution (Supplementary Figure S1). The explanatory variables included in the regression models were child, primary caregiver, and household characteristics as described earlier. Variables with a global Wald-test *p*-value < 0.05 in the univariable models were included in the multivariable models. Scatter plots of residuals against the values of the linear predictor and Q–Q plots were used to assess the linearity, constant variance, and normality of residuals assumptions of linear regression for the multivariable models. Model assumptions were satisfactorily met (Supplementary Figure S2). Statistical significance was set at α = 0.05.

## Results

### Missing data

Data missingness was < 4% in the study. We therefore conducted a complete case analysis for all the analyses reported in the study.

### Characteristics of study participants

Table 1 shows child, primary caregiver, and household characteristics of participants. A total of 2903 children with a mean (SD) age of 15.12 (9.8) months, with approximately 50.2% being females were included in the study. Most children were from households with mothers who had attended at least one ANC visit (98.0%), delivered in a hospital/clinic (97.9%), and had a normal birth weight (≥2,500 g; 91.8%). Additionally, 24.8% of the children were born to mothers who reported experiencing complications during pregnancy with the index child.

**Table 1:** Participants' sociodemographic characteristics.

Characteristic	*N* = 2,903
Child age (months), mean (SD)	15.12 (9.8)
Child gender, *n* (%)	
Female	1,458 (50.2)
Male	1,445 (49.8)
Mother attended any ANC visit, *n* (%)	2,844 (98.0)
Missing	2
Place of delivery, *n* (%)	
Home	62 (2.1)
Hospital/clinic	2,834 (97.9)
Missing	7
Child ever admitted to a hospital, *n* (%)	270 (9.3)
Missing	1
Child birthweight, *n* (%)	
Normal	2,635 (91.8)
Low	236 (8.2)
Missing	32
Mother had problems during pregnancy, *n* (%)	718 (24.8)
Missing	9
Relationship with the child, *n* (%)	
Aunt/uncle	10 (0.3)
Biological father	62 (2.1)
Biological mother	2,782 (95.8)
Brother/sister	2 (0.1)
Grandparent	46 (1.6)
Stepparent	1 (0.0)
Caregiver's age (years), mean (SD)	29.13 (7.0)
Missing	23
Marital status, *n* (%)	
Single	403 (13.9)
Married/cohabiting	2,230 (76.8)
Separated/divorced/widowed	270 (9.3)
Education level, *n* (%)	
Primary	820 (28.2)
Secondary	1,586 (54.6)
Tertiary	497 (17.1)
Positive screen for depressive symptoms, *n* (%)	402 (13.8)
Positive screen for anxiety symptoms, *n* (%)	232 (8.0)
Wealth index, *n* (%)	
Low	707 (24.4)
Middle	1,481 (51.0)
High	715 (24.6)
Settlement type, *n* (%)	
Formal	1,142 (39.3)
Informal	1,761 (60.7)
1	2,524 (88.0)
2+	343 (12.0)
Missing	36
Food insecurity status, *n* (%)	
Secure	671 (23.1)
Mild insecure	464 (16.0)
Moderately insecure	1,668 (57.5)
Severely insecure	100 (3.4)
The household has a child with disability, *n* (%)	54 (1.9)
Missing	11
Home screening score, mean (SD)	10.64 (2.7)

The primary caregivers had a mean (SD) age of 29.13 (7.0) years, with the majority being biological mothers (95.8%). Most caregivers were married/cohabiting (76.8%) and had attained secondary-level education (54.6%). Approximately 60.7% of the study participants were from informal settlements, and 51.0% were from households classified with a middle wealth index status. About 57.5% and 3.4% of the participants were from households categorized as moderately and severely food insecure, respectively (Table 1).

### Nutritional characteristics

Table 2 summarizes the nutritional characteristics of the children in the study. The mean (SD) HAZ and WAZ were −0.90 (1.5) and −0.38 (1.3), respectively. Overall, 22% and 10% of children were classified as stunted and underweight, respectively, with 6.6% having comorbid stunting and underweight.

**Table 2:** Nutritional characteristics of the children included in the study.

Characteristic	*N* = 2,903
Height-for-age *Z*-score (HAZ), mean (SD)	−0.90 (1.5)
Missing	73
Weight-for-age *Z*-score (WAZ), mean (SD)	−0.38 (1.3)
Missing	60
Stunted (HAZ < −2)^a^, *n* (%)	623 (22.0)
Missing	73
Underweight (WAZ < −2)^a^, *n* (%)	284 (10.0)
Missing	60
Stunted and underweight, *n* (%)	189 (6.6)
Missing	34

^a^The proportion of children stunted and underweight was defined as children with HAZ and WAZ scores 2 standard deviations (SD) below the median of the WHO growth reference population.

### Factors associated with stunting

Results from the univariable and multivariable linear mixed effects regression models of factors associated with stunting (HAZ scores) are presented in Table 3. In the multivariable models, a combination of child and household characteristics were significantly associated with stunting. Key child-specific factors included age, gender, and birthweight, while settlement type was a notable household-level determinant. A month's increase in child age was, on average, associated with a 0.04 (95% *CI*: 0.03 to 0.04, *p* < 0.001) decrease in HAZ score, while the effect may seem modest in magnitude, over 24 months, the effect implies nearly a 1 (0.96) SD decline in HAZ scores. Males had, on average, lower HAZ scores by 0.30 (95% *CI*: 0.19 to 0.41, *p* < 0.001) compared to females, while low birthweight (β = −1.19; 95% *CI*: −1.38 to −0.99, *p* < 0.001), residing in informal settlements (β = −0.15; 95% *CI*: −0.27 to −0.04, *p* = 0.010), and severe household food insecurity relative to secure status was associated with lower HAZ scores (β = −0.47; 95% *CI*: −0.78 to −0.15, *p* = 0.004). While we only observed a significant difference in HAZ scores between children from severely food-insecure and secure households, we note that the prevalence of moderate food insecurity was high (57.5%). Although not statistically significant (*p* = 0.065), our assessment of a potential dose-response relationship suggested a trend toward progressively lower HAZ scores with increasing severity of food insecurity (β = −0.06; 95% *CI*: −0.13 to 0.00).

**Table 3:** Results from univariable and multivariable linear mixed effects regression models of factors associated with stunting.

Characteristic	Univariable models		Multivariable model	
	β (95% *CI*)	*P*-value	β (95% *CI*)	*P*-value
Child age (months)	−0.04 (−0.04 to −0.03)	< 0.001	−0.04 (−0.04 to −0.03)	< 0.001
Child gender		< 0.001		
Female	_		_	
Male	−0.30 (−0.41 to −0.19)		−0.30 (−0.41 to −0.19)	< 0.001
Mother attended any ANC		0.845		
No	_			
Yes	0.04 (−0.37 to 0.45)			
Place of delivery		0.514		
Home	_			
Hospital/clinic	0.13 (−0.27 to 0.54)			
Child ever been admitted to a hospital		< 0.001		
No	_		_	
Yes	−0.45 (−0.64 to −0.25)		−0.18 (−0.36 to 0.01)	0.066
Child birthweight		< 0.001		
Normal	_		_	
Low	−1.26 (−1.47 to −1.06)		−1.19 (−1.38 to −0.99)	< 0.001
Mother had problems during pregnancy		0.361		
No	_			
Yes	−0.06 (−0.19 to 0.07)			
Caregiver's age (years)	0.01 (0.00 to 0.01)	0.156		
Marital status		0.035		
Single	_		_	
Married/cohabiting	0.18 (0.01 to 0.35)		0.13 (−0.04 to 0.29)	0.129
Separated/divorced/widowed	0.00 (−0.24 to 0.24)		0.13 (−0.10 to 0.37)	0.257
Education level		0.005		
Primary	_		_	
Secondary	0.17 (0.04 to 0.30)		0.05 (−0.08 to 0.18)	0.419
Tertiary	0.27 (0.09 to 0.44)		0.13 (−0.04 to 0.31)	0.141
Positive screen for depressive symptoms		0.016		
No	_		_	
Yes	−0.20 (−0.37 to −0.04)		−0.06 (−0.23 to 0.10)	0.443
Positive screen for anxiety symptoms		0.820		
No	_			
Yes	−0.02 (−0.24 to 0.19)			
Wealth index		0.002		
Low	_		_	
Middle	0.18 (0.04 to 0.32)		0.12 (−0.02 to 0.26)	0.091
High	0.29 (0.13 to 0.46)		0.14 (−0.04 to 0.31)	0.121
Settlement type		< 0.001		
Formal	_		_	
Informal	−0.23 (−0.35 to −0.12)		−0.15 (−0.27 to −0.04)	0.010
1	_			
2+	0.03 (−0.15 to 0.20)			
Food insecurity status		< 0.001		
Secure	_		_	
Mild insecure	−0.11 (−0.30 to 0.07)		−0.07 (−0.25 to 0.10)	0.416
Moderately insecure	−0.26 (−0.40 to −0.12)		−0.07 (−0.21 to 0.07)	0.315
Severely insecure	−0.49 (−0.83 to −0.16)		−0.47 (−0.78 to −0.15)	0.004
The household has a child living with disability		0.945		
No	_			
Yes	0.01 (−0.41 to 0.44)			
Home screening score	−0.04 (−0.06 to −0.02)	< 0.001	−0.01 (−0.03 to 0.01)	0.395

CI, confidence interval, *n* = 2,799 for the multivariable model. The multivariable model explained approximately 12.7% of the variation in HAZ scores based on the fixed effects. Only variables with a global Wald test *p* < 0.05 in the univariable models were included in the multivariable models. Dash (_) denotes reference group.

### Factors associated with underweight

Child age, gender, hospitalization, birthweight, household wealth status, and settlement type were significantly associated with underweight (WAZ scores) in the multivariable linear mixed effects model (Table 4). A child's increase in age (β = −0.01; 95% *CI*: −0.02 to −0.01, *p* < 0.001), male gender (β = −0.22; 95% *CI*: −0.31 to −0.13, *p* < 0.001), hospitalization (β = −0.17; 95% *CI*: −0.33 to −0.01, *p* = 0.032), and low birthweight (β = −0.94; 95% *CI*: −1.11 to −0.78, *p* < 0.001) were associated with lower WAZ scores. Children from households with a high relative to low wealth index status (β = 0.17; 95% *CI*: 0.03 to 0.31, *p* = 0.015), and those living formal relative to informal settlements (β = 0.14; 95% *CI*: 0.04 to 0.24, *p* = 0.008) had higher WAZ scores, on average.

**Table 4:** Results from univariable and multivariable linear mixed effects regression models of factors associated with underweight.

Characteristic	Univariable models		Multivariable model	
	β (95% *CI*)	*P*-value	β (95% *CI*)	*P*-value
Child age (months)	−0.02 (−0.02 to −0.01)	< 0.001	−0.01 (−0.02 to −0.01)	< 0.001
Child gender		< 0.001		
Female	_		_	
Male	−0.22 (−0.31 to −0.13)		−0.22 (−0.31 to −0.13)	< 0.001
Mother attended any ANC		0.814		
No	_			
Yes	−0.04 (−0.38 to 0.30)			
Place of delivery		0.044		
Home	_		_	
Hospital/clinic	0.33 (0.01 to 0.65)		0.32 (−0.01 to 0.65)	0.058
Child ever been admitted to a hospital		< 0.001		
No	_		_	
Yes	−0.35 (−0.51 to −0.19)		−0.17 (−0.33 to −0.01)	0.032
Child birthweight		< 0.001		
Normal	_		_	
Low	−0.99 (−1.16 to −0.82)		−0.94 (−1.11 to −0.78)	< 0.001
Mother had problems during pregnancy		0.939		
No	_			
Yes	0.00 (−0.11 to 0.10)			
Caregiver's age (years)	0.00 (0.00 to 0.01)	0.552		
Marital status		0.143		
Single	_			
Married/cohabiting	0.10 (−0.03 to 0.24)			
Separated/divorced/widowed	−0.01 (−0.21 to 0.18)			
Education level		0.058		
Primary	_			
Secondary	0.06 (−0.05 to 0.16)			
Tertiary	0.17 (0.03 to 0.31)			
Positive screen for depressive symptoms		0.002		
No	_		_	
Yes	−0.22 (−0.35 to −0.08)		−0.12 (−0.26 to 0.03)	0.125
Positive screen for anxiety symptoms		0.035		
No	_		_	
Yes	−0.18 (−0.36 to −0.01)		−0.09 (−0.28 to 0.09)	0.328
Wealth index		< 0.001		
Low	_		_	
Middle	0.11 (0.00 to 0.23)		0.06 (−0.05 to 0.18)	0.285
High	0.25 (0.12 to 0.39)		0.16 (0.03 to 0.30)	0.018
Settlement type		< 0.001		
Formal	_		_	
Informal	−0.21 (−0.31 to −0.10)		−0.14 (−0.24 to −0.04)	0.008
1	_			
2+	−0.07 (−0.21 to 0.07)			
Food insecurity status		0.297		
Secure	_			
Mild insecure	−0.06 (−0.21 to 0.09)			
Moderately insecure	−0.10 (−0.21 to 0.01)			
Severely insecure	−0.18 (−0.45 to 0.09)			
The household has a child living with disability		0.185		
No	_			
Yes	−0.23 (−0.57 to 0.11)			
Home screening score	0.00 (−0.02 to 0.01)	0.597		

CI, confidence interval, *n* = 2,804 for the multivariable model. The multivariable model explained approximately 7.9% of the variation in WAZ scores based on the fixed effects. Dash (_) denotes reference group.

## Discussion

This study investigated stunting and underweight conditions among children aged 0–3 years in Nairobi City County, Kenya. The findings revealed that 22.0% of the children were stunted, 10.0% were underweight, and 6.6% had both conditions. The analysis revealed an intricate interplay of child, maternal, and socioeconomic factors influencing child nutritional outcomes. The key factors associated with stunting and underweight identified in the multivariable models included the child's age and gender, low birth weight, type of settlement, and food insecurity.

A child's age was significantly associated with both stunting and underweight, with increasing age associated with lower HAZ and WAZ scores, respectively. This trend likely reflects the cumulative effect of prolonged exposure to risk factors such as inadequate complementary feeding, recurrent infections, and a challenging environment after the protective effects of exclusive breastfeeding diminish (29). In many low-resource urban settings, complementary feeding practices are often suboptimal, characterized by late introduction, poor dietary diversity, and low energy density, which compromises child growth (29). Additionally, older infants and toddlers have increased mobility and contact with contaminated environments, heightening the risk of infections such as diarrhea, which further impairs nutrient absorption and appetite (30). While the magnitude of the effect of age on weight and height was small, its cumulative effect over time can result in substantial deficits in HAZ and WAZ scores.

Sex differences were evident, with boys exhibiting significantly lower height-for-age and weight-for-age *z*-scores (WAZ) compared to girls. This finding is consistent with evidence from studies across SSA (31, 32). Boys may have higher basal metabolic demands and faster growth velocity during infancy, which increases their nutritional requirements and renders them more vulnerable when these needs are unmet (33). This vulnerability may be linked to higher susceptibility to infections and a greater sensitivity to environmental stressors among boys in early infancy (33). These sex-based disparities underscore the importance of tailoring nutrition interventions that account for biological and contextual vulnerabilities, ensuring equitable care and nutrition for boys and girls during the critical first 1,000 days of life.

Consistent with previous research (3, 34), this study reinforces the influence of factors established at or before birth on child nutritional outcomes. Low birth weight was significantly associated with both stunting and underweight, with affected children exhibiting markedly lower height-for-age and weight-for-age *Z*-scores. This association may underscore the critical role of intrauterine growth and maternal health in shaping early nutrition and growth trajectories. Low birth weight often results from maternal undernutrition, infections during pregnancy, inadequate antenatal care, and limited access to healthcare services during pregnancy (3). Infants born small are more susceptible to infections, have reduced nutrient reserves, and may experience delayed growth catch-up, increasing the risk of chronic undernutrition (3). Studies across SSA consistently show that children with low birth weight are two to three times more likely to be stunted or underweight compared to those with normal birth weight (34). These findings highlight the need for interventions focused on maternal nutrition, infection prevention, and improved antenatal care to reduce childhood undernutrition.

Living in an informal settlement was associated with both stunting and underweight, reflecting how the structural and environmental disadvantages inherent to these settings may heighten the risk of undernutrition. Poor water and sanitation infrastructure, inadequate waste management, and high population density common in informal settlements create conditions for frequent infections that impair nutrient absorption and compromise growth (35). Limited access to health services and preventive care further reduces opportunities for timely illness management, exacerbating the risk of undernutrition (35). Research has shown that urban poverty, characterized by poor environmental conditions and limited access to basic services, significantly contributes to growth faltering in populations living in urban informal settlements and often results in worse nutritional outcomes than those observed in rural areas (36). Addressing these disparities requires multi-sectoral interventions, including improvements in water, sanitation, and hygiene (WASH), access to quality healthcare, and broader social protection measures to mitigate structural poverty and enhance overall living conditions.

Severe food insecurity was significantly associated with increased stunting. In food-insecure households, coping strategies often include reducing meal frequency, decreasing portion sizes, or relying on cheaper, energy-dense staple diets with low micronutrient quality; practices that limit children's dietary diversity and micronutrient intake (37). According to a study conducted in Kenya, food insecurity also influences feeding practices, and caregivers may delay initiating complementary feeding, dilute nutrient-dense foods, or reduce animal-source foods to stretch limited resources during infancy (38). Prolonged dietary inadequacy during early life perpetuates a malnutrition cycle, weakened immunity, and reduced attainment of developmental potential (39). Although a significant association was only observed for the comparison of severely food-insecure vs. food-secure households, our dose-response analysis, although not statistically significant (*p* = 0.065), suggested a trend toward progressively lower HAZ scores with increasing severity of food insecurity. This potential dose-response relationship may call for household-level strategies to improve food availability and accessibility alongside nutrition-sensitive interventions like kitchen gardening, planting vegetables in sacks or containers, and small-scale livestock rearing (e.g., poultry or rabbits), given the high prevalence of food insecurity in our sample.

Hospital admission was associated with underweight, with children who had ever been hospitalized showing lower weight-for-age *Z*-scores. This is biologically plausible since severe illness (that requires admission) suppresses appetite, increases metabolic demands, and can impair nutrient absorption, factors which can lead to acute weight loss and hinder catch-up growth (40). Recurrent or severe diarrhoeal illness is a known driver of growth faltering due to fluid and nutrient losses, intestinal inflammation, and postinfectious malabsorption (41). Elsewhere, a retrospective review of records from Garissa County Referral Hospital in Kenya identified diarrhea as one of the most common reasons for admission among children aged 6–59 months, and it was linked with other infections and undernutrition (42). Episodes of respiratory tract infection (RTI), such as pneumonia, which are common in young children, are also associated with reduced intake and elevated energy expenditure from labored breathing (43). An epidemiological study in Nairobi's informal settlements has shown links between cough/rapid breathing and wasting in young children, illustrating the growth impact of acute respiratory morbidity in high-risk urban environments (36). These findings highlight the need for integrated child health strategies that combine prompt treatment of common childhood illnesses with nutritional rehabilitation and caregiver education on feeding during and after illness. Strengthening infection prevention measures, including improved hygiene, vaccination coverage, and timely access to healthcare, is also critical to reducing illness-related undernutrition. However, it should be noted that in our model, hospitalization may act as a mediator in the pathway between socioeconomic disadvantage (wealth status) and the underweight presentation.

The multivariable linear regression analysis revealed that there was an association between household wealth status and underweight, with children from wealthier households having higher weight-for-age *z*-scores. This finding aligns with evidence from other SSA countries, where socioeconomic status strongly influences child nutrition (44, 45). Wealthier households are more likely to access diverse, nutrient-rich food, improved living conditions, and timely healthcare services, all of which collectively promote optimal child growth. Conversely, households in the lowest wealth categories often face food insecurity, inadequate sanitation, and overcrowding, which increase infection risk and impair nutrient absorption, perpetuating undernutrition.

Although several caregiver and household-level factors were significant in univariable analyses, none remained associated with stunting or underweight after multivariable adjustment. For stunting, maternal education, caregiver depressive symptoms, and the home environment score showed significant associations in univariable models but lost significance after adjustment, suggesting a complex interplay between variables. For underweight, caregiver depressive symptoms were associated with lower WAZ scores in univariable analyses but were no longer significant in the adjusted model, perhaps indicating a pronounced effect of biological factors such as age, sex, and birthweight, as well as structural conditions including household wealth and settlement type. The lack of associations for psychosocial and caregiving variables may suggest that, in this urban context, structural and biological vulnerabilities exert a stronger influence on child anthropometric status when evaluated alongside one another. These null findings highlight the importance of multivariable analyses in assessing the determinants of stunting and underweight.

A key feature of the urban nutritional paradox is that food insecurity often persists alongside better access to essential services in urban areas (46). Our findings show that food insecurity was associated with stunting but not underweight. In urban settlements, food insecurity may be intermittent rather than absolute, with households relying on informal markets, small daily food purchases, or social support mechanisms that buffer short-term weight loss but do not prevent cumulative linear growth faltering (47). Differences in the pathways leading to chronic and acute undernutrition may explain this, since linear growth is more sensitive to sustained inadequacies in diet quality and repeated infections, while body weight may be preserved in the short term through minimal caloric sufficiency (48). In such contexts, children may consume enough energy-dense staple foods to maintain weight but lack the dietary diversity and micronutrients necessary for optimal linear growth. Conversely, the wealth index was associated with underweight but not stunting, suggesting that current household economic capacity may influence short-term nutritional status through access to food, healthcare, and illness management, while being insufficient to offset long-standing environmental and biological risks that shape linear growth. These patterns are likely shaped by broader urban dynamics, including reliance on unstable, cash-based livelihoods, limited access to affordable nutrient-dense foods within informal food environments, and high population mobility that may disrupt caregiving and feeding practices (49). Together, these factors underscore the complexity of child nutrition in rapidly urbanizing settings, where structural constraints may sustain chronic undernutrition despite proximity to health services.

## Strengths and limitations

The study benefited from a large sample size drawn from both formal and informal settlements across the entire Nairobi County, allowing for comparisons across diverse urban settings while maintaining precision in the estimates. This is one of the few studies that have focused on the critical growth window, often masked in under-five analyses. Anthropometric measurements were taken following the WHO standardized protocol, minimizing measurement error and enhancing data accuracy. Validated study instruments were used to assess key psychosocial and household factors, including the PHQ-9 and GAD-7 for caregiver mental health and the HFIAS for household food insecurity, strengthening the reliability of study findings.

However, the study had several limitations. First, its cross-sectional design limits the ability to infer causal relationships. Some variables, such as ANC attendance, illness history, and pregnancy complications, were self-reported by caregivers, introducing potential recall and social desirability biases. Furthermore, the study did not capture detailed information on dietary intake, severity of childhood illnesses, or environmental contamination, which could result in low predictive power and low outcome variance explained by the predictor variables (Adjusted *R*^2^) of the regression models. Indeed, although the study identified statistically significant factors associated with HAZ and WAZ, the adjusted *R*^2^ values of 12.7% and 7.9% suggested that substantial variability remained unexplained in HAZ and WAZ, respectively. Future studies should consider additionally including these variables that were not captured in the present study.

## Conclusion

This study identified key factors associated with stunting and underweight among a sample of children under 3 years of age in Nairobi. The findings highlight that the drivers of undernutrition in this urban setting are multifaceted, stemming from biological vulnerabilities that are aggravated by socioeconomic inequalities. Addressing undernutrition in this context requires more than blanket interventions. Targeted strategies that improve maternal and child health during the perinatal period, improve household food security, and strengthen WASH systems are essential to ensure children not only survive but also attain optimal growth and development. While this study provides valuable cross-sectional insights, longitudinal research is needed to establish causal pathways and better understand the dynamic interaction among biological, environmental and social determinants of child nutrition over time.

## Data Availability

The datasets presented in this article are not readily available because data will be made available upon reasonable request following the Aga Khan University data sharing policies and procedures. Data requests should be addressed to the corresponding author. Requests to access the datasets should be directed to eunice.njoroge@aku.edu.

## Supplementary material

Supplementary Figure S1 Histograms and density plots showing the distribution of height-for-age *Z*-scores (HAZ) and weight-for-age *Z*-scores (WAZ) in our sample.

Supplementary Figure S2 Scatter plot of residuals against values of the linear predictor and Q–Q plots of residuals from the multivariable linear regression model of factors associated with stunting and underweight.
