# Supplementary material for: Factors associated with stunting and underweight indices among children 0–3 years in Nairobi, Kenya: a cross-sectional study
Source: Front Nutr. 2026 May 11;13:1793821. doi: 10.3389/fnut.2026.1793821 (PMC13200522; doi:10.3389/fnut.2026.1793821)
Supplement: Supplementary Figure S1 — Histograms and density plots showing the distribution of height-for-age Z-scores (HAZ) and weight-for-age Z-scores (WAZ) in our sample. [file Image_1.pdf]

## Supplementary Figures

**Factors associated with stunting and underweight indices among children 0-3 years in Nairobi, Kenya: a cross-sectional study**

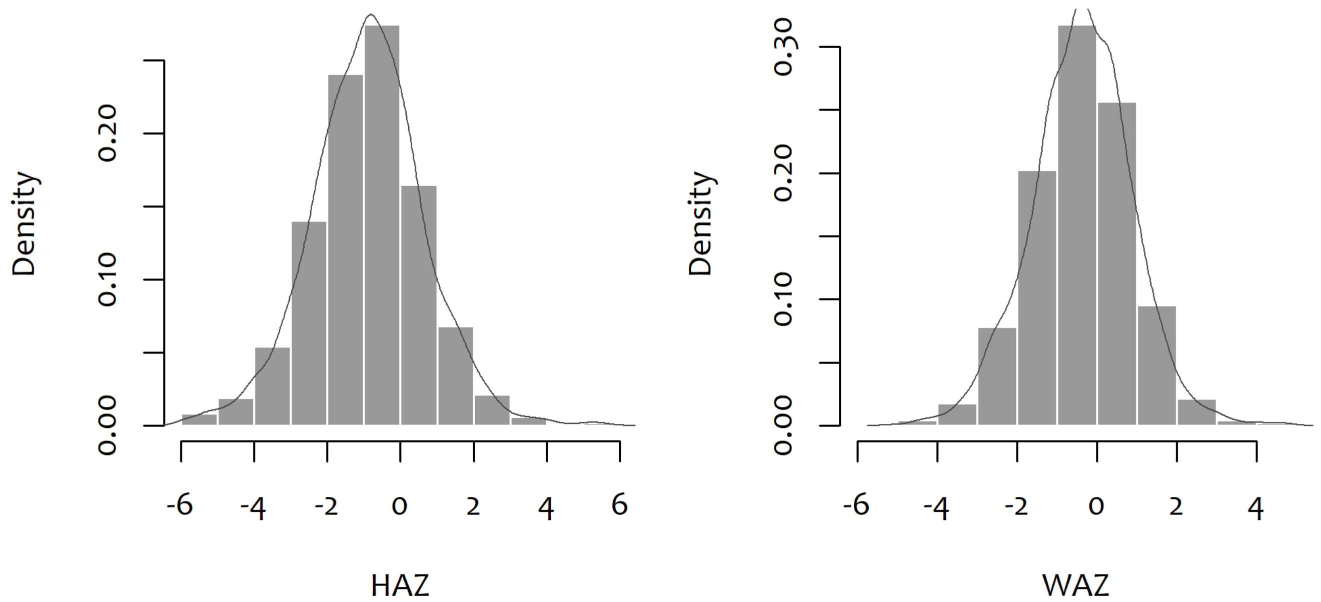


Supplementary Figure 1: Distribution of height-for-age Z-scores (HAZ) and weight-for-age Z-scores (WAZ) in our sample.

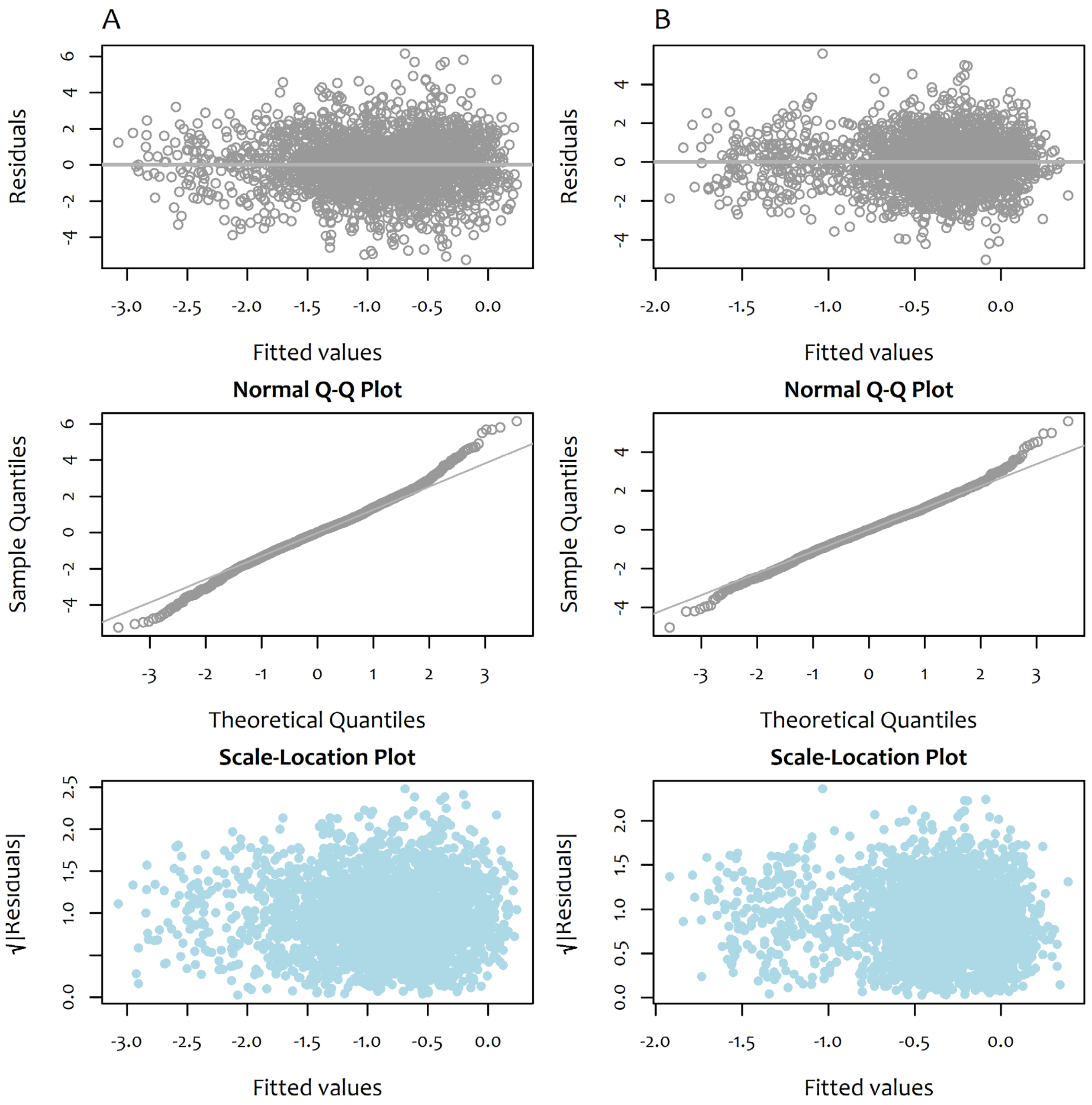


Supplementary Figure 2: Plots showing the assessment of the linear mixed effects model assumption assessment; column A is for stunting and column B for underweight. The first row shows residuals vs values of the linear predictor, the second row shows Q-Q plots of residuals, and the third row shows the square-root of absolute residuals against the fitted values.
